# Expression of Concern: In vivo NCL targeting affects breast cancer aggressiveness through miRNA regulation

**DOI:** 10.1084/jem.2012095009272022e

**Published:** 2022-10-03

Based on an investigation by the College of Medicine Investigation Committee (the COMIC) at The Ohio State University, and *JEM*’s review, *JEM* is issuing this Expression of Concern regarding Figures 1 E and 1 F from Pichiorri et al. (2013) *J. Exp. Med.* 210(5):951–68 and Correction Pichiorri et al. (2017) *J. Exp. Med.* 214(5):1557.

Vol. 210, No. 5 | https://doi.org/10.1084/jem.20120950 | April 22, 2013

Vol. 214, No. 5 | https://doi.org/10.1084/jem.2012095001172017c | January 19, 2017
